# Exploring the adsorption behavior of molecular hydrogen on CHA-zeolite by comparing the performance of various force field methods

**DOI:** 10.1039/d3ra04262f

**Published:** 2023-10-23

**Authors:** Muhammad Tariq Aziz, Syed Ali Raza Naqvi, Muhammad Ramzan Saeed Ashraf Janjua, Manawwer Alam, Waqas Amber Gill

**Affiliations:** a Department of Chemistry, Government College University Faisalabad Faisalabad 38000 Pakistan draliraza@gcuf.edu.pk Janjua@gcuf.edu.pk Dr_Janjua2010@yahoo.com; b Department of Chemistry, College of Science, King Saud University Riyadh 11451 Saudi Arabia; c Departamento de Química Física, Universidad de Valencia Avda Dr Moliner, 50, E-46100 Burjassot Valencia Spain wagill@alumni.uv.es

## Abstract

Molecular hydrogen (H_2_) adsorption plays a crucial role in numerous applications, including hydrogen storage and purification processes. Understanding the interaction of H_2_ with porous materials is essential for designing efficient adsorption systems. In this study, we investigate H_2_ adsorption on CHA-zeolite using a combination of density functional theory (DFT) and force field-based molecular dynamics (MD) simulations. Firstly, we employ DFT calculations to explore the energetic properties and adsorption sites of H_2_ on the CHA-zeolite framework. The electronic structure and binding energies of H_2_ in various adsorption configurations are analyzed, providing valuable insights into the nature of the adsorption process. Subsequently, force field methods are employed to perform extensive MD simulations, allowing us to study the dynamic behavior of H_2_ molecules adsorbed on the CHA-zeolite surface. The trajectory analysis provides information on the diffusion mechanisms and mobility of H_2_ within the porous structure, shedding light on the transport properties of the adsorbed gas. Furthermore, the combination of DFT and MD results enables us to validate and refine the force field parameters used in simulations, improving the accuracy of the model, and enhancing our understanding of the H_2_–CHA interactions. Our comprehensive investigation into molecular hydrogen adsorption on CHA-zeolite using density functional theory and molecular dynamics simulations yields valuable insights into the fundamental aspects of the adsorption process. These findings contribute to the development of advanced hydrogen storage and separation technologies, paving the way for efficient and sustainable energy applications.

## Abstract 2

We employed various force field methods to identify the interaction potential that provides the best match for this particular kind of adsorption .

## Graphical abstract

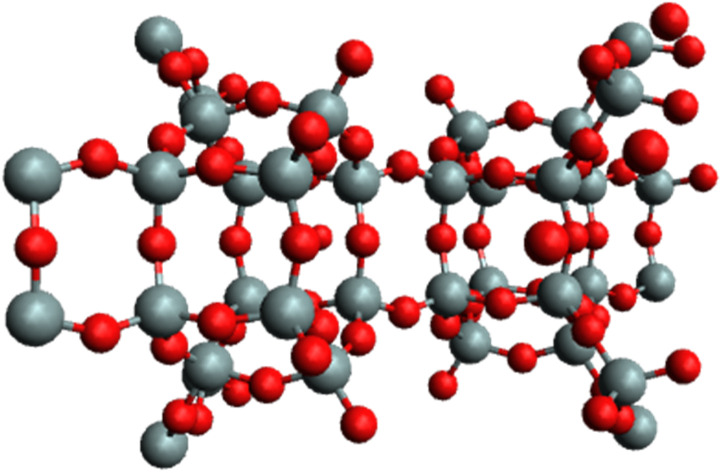

## 1. Introduction

CHA-zeolite, also known as chabazite zeolite, is a highly adaptable and extensively utilized adsorbent material with significant applications in numerous fields.^1^ It is the material of choice for adsorption processes due to its unique structural characteristics, exceptional thermal stability, and high surface area.^2^ The crystal structure of CHA-zeolite is composed of interconnected channels and cages, which provide a large surface area for adsorption.^3^ CHA-zeolite's framework contains a high concentration of acidic sites, making it suitable for a variety of adsorption processes.^4^ CHA-zeolite's adsorption capacity can be explained by multiple mechanisms, including physisorption and chemisorption.^5^

Chemisorption entails the formation of chemical bonds between the adsorbate and the acidic sites present in the zeolite framework.^6^ The combination of these mechanisms permits CHA-zeolite to effectively absorb a vast array of substances.^7^ In gas separation and purification processes, CHA-zeolite is widely employed.^8^ It is appropriate for separating molecules of various sizes and polarities due to its high selectivity and adsorption capacity.^9^ Physisorption of CHA-zeolite refers to the non-covalent, weak interactions between gas molecules (*e.g.*, H_2_, CO_2_) and the surface of the zeolite framework.^10^ These interactions are primarily driven by van der Waals forces,^5^ enabling reversible adsorption and desorption processes and making CHA-zeolite a promising material for gas storage and separation applications.

CHA-zeolite, for instance, can be used to remove CO_2_ from natural gas or flue gas streams, thereby contributing to the reduction of greenhouse gas emissions.^11^ Due to its large surface area and well-defined pores, CHA-zeolite is an effective catalyst support material.^12^ Various catalytic processes, such as petrochemical refinement, fine chemical synthesis, and environmental remediation, have utilized it.^13^ The acidic sites within the framework of zeolite enhance catalytic activity and selectivity, resulting in enhanced reaction efficiency.^14^ In adsorption heat pump applications, CHA-zeolite has the potential to be used for heating, cooling, and energy storage.^15^ Using the adsorption–desorption cycle of CHA-zeolite, heat can be conveyed and stored efficiently, providing a sustainable alternative to conventional heating and cooling systems.^16^

Several investigations have examined the adsorption of H_2_ on CHA-zeolite utilizing various experimental and computational techniques. Understanding the adsorption behavior, capacity, and kinetics of H_2_ on CHA-zeolite, as well as the factors influencing the adsorption process, has been the focus of these studies. Based on grand canonical Monte Carlo simulations^17^ of CHA-zeolite's H_2_ adsorption properties, CHA-zeolite has a high H_2_ adsorption capacity, with an adsorption energy that increases with increasing Si/Al ratio. Additionally, the simulations demonstrated that H_2_ molecules preferentially adsorb in the sodalite cages of the CHA-zeolite framework. The effect of temperature,^18^ utilized *in situ* infrared spectroscopy to probe the interaction between H_2_ molecules and CHA-zeolite at different temperatures.

The results showed that H_2_ adsorption on CHA-zeolite is an exothermic process, and increasing the temperature leads to a decrease in the adsorption capacity.^19^ At varied temperatures and pressures, the H_2_ adsorption capacity^20^ of CHA-zeolite has been examined. The H_2_ adsorption increased with increasing pressure, and the Si/Al ratio and pore size of CHA-zeolite significantly influenced its adsorption capacity.^21^ It was determined that CHA-zeolite has excellent stability and recyclability for applications involving H_2_ adsorption.^22^ The research demonstrated that CHA-zeolite is a promising material for H_2_ storage and purification.^23–27^ The effect of framework flexibility^28^ on H_2_ adsorption in CHA-zeolite was investigated using molecular simulations and density functional theory calculations.^29,30^ Their findings demonstrated that the flexibility of the CHA-zeolite framework influences the energy and capacity of H_2_ adsorption. Controlling the framework's flexibility may be a viable strategy for enhancing the H_2_ adsorption properties of CHA-zeolite.^31^

Utilizing simulations of molecular dynamics (MD)^32^ to investigate the adsorption behavior of H_2_ on CHA-zeolite. The purpose of this study is to present a set of systematic potentials that characterize the interactions between molecular hydrogen and CHA-zeolite. These potentials establish a balance between precision and simplicity, making them suitable for simulations of molecular dynamics. To compare the efficacy of the proposed force fields, molecular dynamics simulations will be calculated using *NVE* (number of particles (*N*), volume (*V*), and total energy (*E*)) ensemble.^33–35^ In addition, we will use precise techniques, such as density functional theory (DFT), to predict the preferable interaction sites of gas molecules on CHA-zeolite and to determine the optimal orientation of molecular hydrogen.

In Section 2, we will describe the computational details utilized in this investigation. The analysis of the derived potential energy surface will be the focus of Section 3, while Section 4 will present the MD simulation. In the final section, Section 5, a summary of the study's findings will be presented.

## 2. Computational details

The widely used Lennard-Jones (LJ) potential is often employed to describe the adsorption of gases on graphene's, metal organic frame works and zeolites.^36,37^ However, the LJ potential is known to have deficiencies at both short and long ranges.^38–40^ To overcome these limitations, Pirani *et al.* proposed an Improved Lennard-Jones (ILJ)^41–43^ potential that addresses the shortcomings of the LJ model. The ILJ potential introduces an additional parameter, enhancing its flexibility and accuracy. Through careful parameter selection, the ILJ potential proves particularly valuable for molecular dynamics simulations, especially for non-covalent interactions.^41,42^ Its accuracy makes it a useful tool for accurately representing the adsorption behavior of gases on CHA-zeolite. In many cases, electrostatic contributions to the interaction are incorporated using a coulombic expression, where partial charges are assigned to the system.^44,45^ The ILJ interaction potentials utilized in this study were derived from first principles calculations performed at the dispersion-corrected density functional theory level.^46^

Specifically, the B97D functional,^47^ which incorporates an empirical dispersion term, was employed. The B97D functional is recognized as a robust approach among semi-empirical generalized gradient approximation (GGA) functionals, offering efficiency and precision in studying large systems that interact *via* dispersion forces.^48^ While the B97D functional has demonstrated its value in accurately describing non-covalent interactions,^49–52^ it is important to note that its performance may not be universally reliable for all systems.

In this study, we utilize the Improved Lennard-Jones (ILJ) potential to construct a series of force fields suitable for assessing van der Waals interactions, particularly within the CHA-zeolite and molecular hydrogen system. Additionally, we explore the impact of the electrostatic component on the force field parameters by incorporating various charge schemes such as Hirshfeld,^53^ CHelpG,^54^ MBS (Minimum Basis Set),^55^ MK (Merz–Kollman),^56^ NBO (Natural Bonding Orbitals),^57^ evaluating their effectiveness and feasibility. To account for the fact that CHA-zeolite is an infinite molecule, we employed truncated system for the single point energy calculations. This model was designed to be large enough to capture the essential interactions. All monomers were initially optimized at the B3LYP/6-31G** level.^58,59^

The distances of the cell matrix are 13.675 Å, 6.8375 Å, 11.8429 Å and 14.767 Å, respectively, for CHA-zeolite. The cell parameters are *A* = 13.675 Å with *α* = 90°, *B* = 13.675 Å with *β* = 90° and *C* = 14.767 Å with *γ* = 120° and with the cell volume 2391.53960 Å^3^. Subsequently, these optimized structures were treated as rigid in all subsequent interaction energy calculations.

**Fig. 1:**
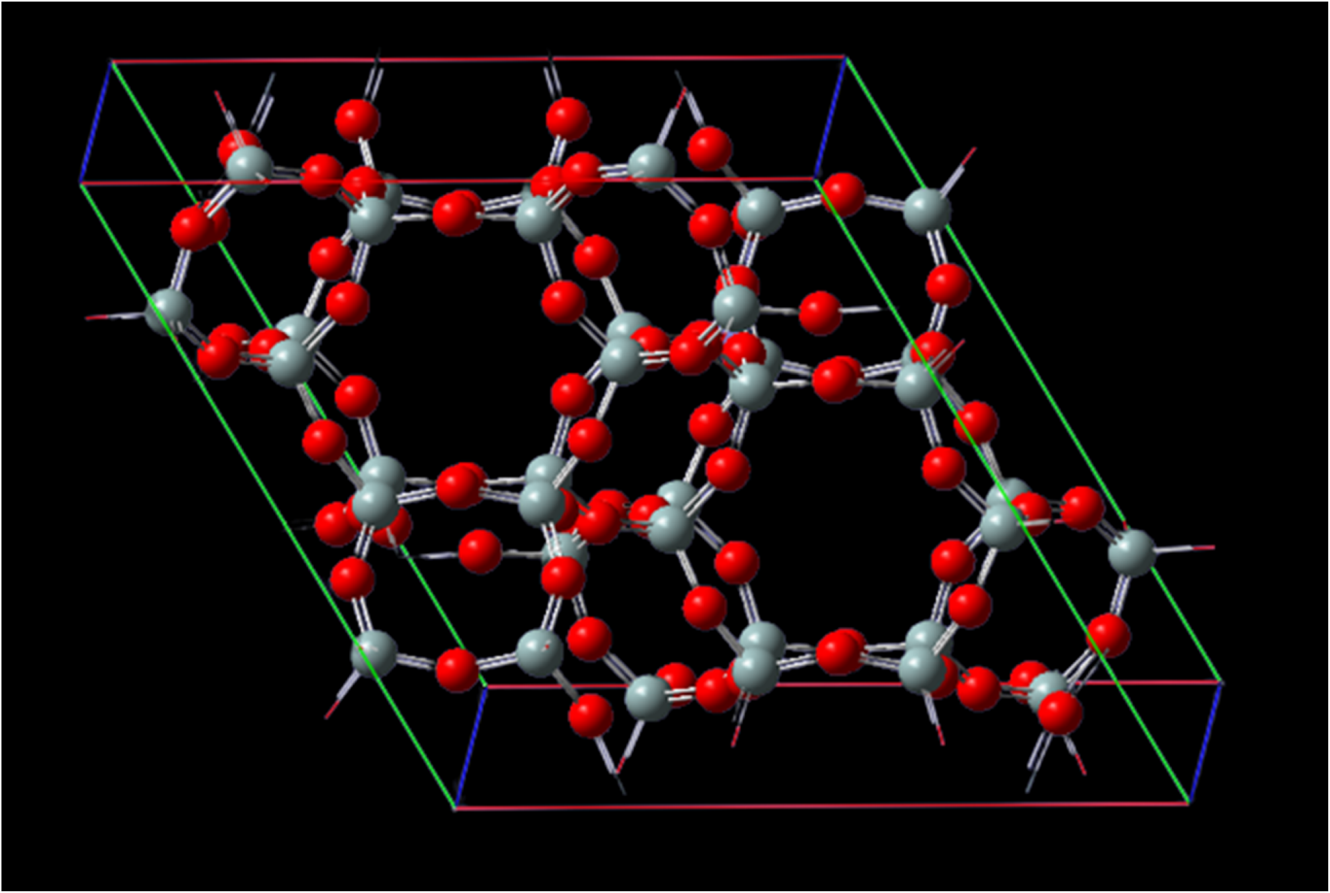
Unit cell matrix of CHA-zeolite are 13.675 Å, 6.8375 Å, 11.8429 Å and 14.767 Å, respectively. The cell parameters are *A* = 13.675 Å with *α* = 90°, *B* = 13.675 Å with *β* = 90° and *C* = 14.767 Å with *γ* = 120° and with the cell volume 2391.53960 Å^3^.

Fig. 1 represents, in crystallography, a crystal lattice is often described using a unit cell, which is the smallest repeating unit that represents the entire crystal structure. The unit cell is defined by three parameters: the lengths of its edges (*A*, *B*, and *C*) and the angles between them (*α*, *β*, *γ*). *A*, *B*, and *C*, these parameters represent the lengths of the edges of the unit cell. They define the size of the unit cell in each direction. Typically, these values are given in angstroms (Å) or picometers (pm). *α*, *β*, *γ* these angles represent the angles between the edges of the unit cell. They define the shape and symmetry of the unit cell. *α* is the angle between *B* and *C*, *β* is the angle between *A* and *C*, and *γ* is the angle between *A* and *B*. These angles are measured in degrees. CHA-zeolite is a specific type of zeolite with a characteristic framework structure consisting of interconnected channels and cages. The unit cell parameters (*A*, *B*, and *C*) and angles (*α*, *β*, *γ*) describe the arrangement of the atoms within this crystal lattice. By knowing the values of *A*, *B*, *C*, alpha, beta, and gamma, one can determine the size, shape, and symmetry of the CHA-zeolite crystal structure. These parameters provide important information about the crystallographic properties and can be used to understand various physical and chemical properties of CHA-zeolite.

The energy calculations for the molecular hydrogen over CHA-zeolite system, were performed using Gaussian 09 (ref. 60) and the EMSL Basis Set Exchange. The calculations were based on the B97D/TZV2P level.^61^ This specific combination of the B97D functional and the split-valence triple-zeta basis set with two polarizations (TZV2P) has been previously determined as adequate for accurately representing polarization effects in comparable systems. A total of 100 random orientations^62^ for the H_2_ molecule were generated by fixing CHA-zeolite. Each orientation was then meticulously scanned for intermolecular distances ranging from 2.5 to 12.0, with a focus on sampling near the equilibrium distance. There was a total of 25 distances considered, which encompassed all 100 relative orientations. Using the B97D/TZV2P method, the interaction energy for each of the 2500 conformations was then calculated.

## 3. Potential energy surface

It is common to divide a potential energy surface into distinct components, such as electrostatic and non-electrostatic forces, when constructing one. For the potential energy surface of H_2_ over CHA-zeolite, electrostatic forces result from coulombic interactions between charged particles within molecules, while non-electrostatic forces result from van der Waals interactions between neutral particles. The total potential energy can be expressed as the sum of electrostatic and non-electrostatic contributions if these components are treated separately, and their separability is assumed. This assumption simplifies the construction procedure and facilitates the independent management of each contribution.

To facilitate efficient calculation of potential energy contributions, it is assumed that both parts of the potential energy surface can be represented as sums over *A* and *B* molecule centres. The interactions between two molecules can be described in a straightforward and computationally efficient manner by totaling the contributions from each atom or centre in both molecules. It is essential to observe, however, that these assumptions may not apply to all molecular systems, and that certain circumstances may necessitate the use of advanced methods.1
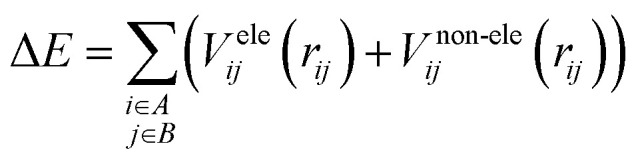


There are different ways to describe the resulting interactions between molecules, which can include coulombic sums over assigned atomic charges. The coulombic sum is given by2
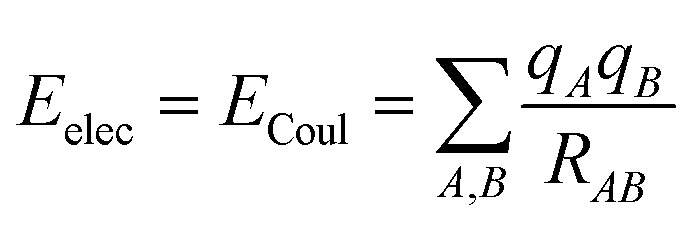


In the potential energy surface, distinct contributions, such as electrostatic and non-electrostatic forces, can be identified. Coulombic interactions between charged particles within the molecules generate electrostatic forces, whereas van der Waals forces between inert particles are responsible for the non-electrostatic forces.

3 *V*_tot_(*R*) = *V*_nelec_(*R*) + *V*_elec_(*R*) = *V*_ILJ_(*R*) + *V*_Coul_(*R*)

In molecular simulations, the ILJ potential is used as a model to characterize the non-electrostatic portion of the potential energy surface. In molecular simulations, non-electrostatic interactions are typically attributed to van der Waals forces caused by fluctuations in the electron cloud encircling atoms and molecules.4
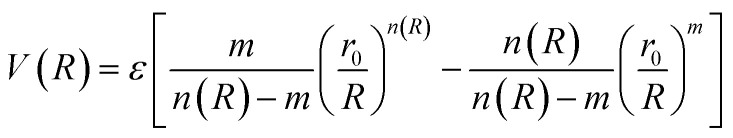
where5
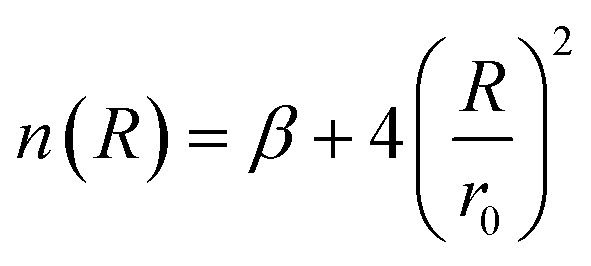
*ε* is the well depth of the dissociation curve depicted by the ILJ potential, while *r*_0_ is its position. On the other hand, *β* is a dimensionless parameter that adds extra flexibility.

In Table 1, we can observe that the *q*_O_ “Fitted” value of 0.652 e^−^ and the “MBS” value of 0.625 e^−^ are relatively close to each other compared to the other values. The difference between these two values is relatively small, suggesting a similarity in their estimation of the charge associated with the oxygen (O) atoms. *ε*_SiH_ represents that the “Fitted” value of 0.127 kJ mol^−1^ and the “MK” value of 0.107 kJ mol^−1^ are relatively close to each other compared to the other values. The difference between these two values is relatively small, indicating a similarity in their estimation of the well-depth or interaction energy associated with the silicon–hydrogen (Si–H) interaction. *r*_SiH_ shows that the “Fitted” value of 3.426 Å and the “MBS” value of 3.445 Å are relatively close to each other compared to the other values. The difference between these two values is relatively small, suggesting a similarity in their estimation of the equilibrium bond length between silicon (Si) and hydrogen (H) atoms (*r*_SiH_). *ε*_OH_ shows that the “Fitted” value of 0.927 kJ mol^−1^ and the “NBO” value of 0.929 kJ mol^−1^ are relatively close to each other compared to the other values. The difference between these two values is relatively small, indicating a similarity in their estimation of the well-depth or interaction energy associated with the oxygen–hydrogen (O–H) interaction (*ε*_OH_). *r*_OH_ shows that the “Fitted” value of 3.782 Å and the “MBS” value of 3.798 Å are relatively close to each other compared to the other values. The difference between these two values is relatively small, suggesting a similarity in their estimation of the equilibrium bond length between oxygen (O) and hydrogen (H) atoms (*r*_OH_). The Lennard-Jones potential can be utilized to investigate the adsorption of molecular hydrogen (H_2_) over CHA-zeolite. By employing the Lennard-Jones potential, we can model the intermolecular interactions between H_2_ molecules and the zeolite surface. This potential describes both the attractive and repulsive forces between molecules as a function of their separation distance. Through simulations and calculations based on the Lennard-Jones potential, it is possible to estimate the adsorption capacity and adsorption energy of H_2_ over CHA-zeolite. These parameters play a crucial role in understanding and optimizing the adsorption process.

**Table 1:** To obtain Lennard-Jones (LJ) potentials for the H_2_/CHA-zeolite, the B97D/TZV2P calculations are used to fit certain parameters

Parameters	Fitted	Hirshfeld	CHelpG	MBS	MK	NBO
*q*_O_ (e^−^)	0.652	0.948	0.978	0.625	0.575	0.925
*ε*_SiH_ (kJ mol^−1^)	0.127	0.129	0.279	0.325	0.107	0.452
*r*_SiH_ (Å)	3.426	3.741	3.562	3.445	3.821	3.625
*ε*_OH_ (kJ mol^−1^)	0.927	0.953	0.966	0.973	0.998	0.929
*r*_OH_ (Å)	3.782	3.841	3.963	3.798	3.948	3.891
*r* ^2^	0.995217	0.996158	0.997182	0.994736	0.998674	0.993256
MAE (kJ mol^−1^)	0.582046	0.425981	0.532744	0.554732	0.478521	0.462574

**Fig. 2:**
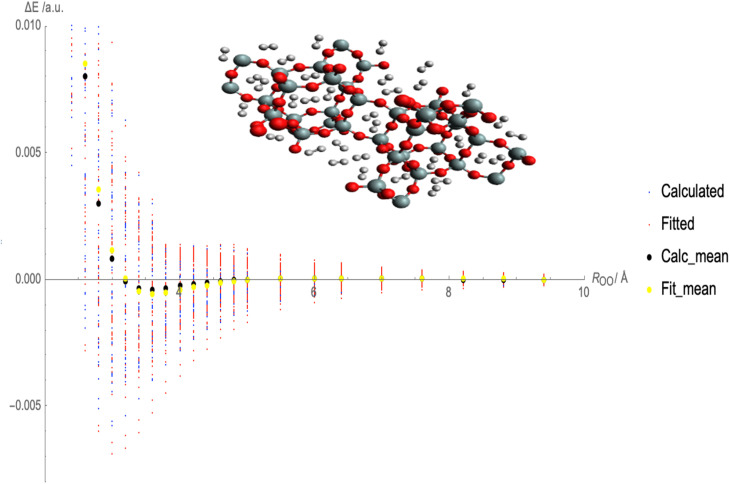
Fitted Lennard-Jones (LJ) potential parameters with charge schemes by using random interaction energies over distances of molecular hydrogen and CHA-zeolite, blue dots show calculated interaction energies, red dots show fitted interaction energies, black dots show calculated average interaction energies and yellow dots show fitted average interaction energies.

Fig. 2 illustrates the fitted Lennard-Jones (LJ) potential parameters for the interaction between molecular hydrogen (H_2_) and CHA-zeolite utilizing various charge schemes. The *x*-axis represents the distances between H_2_ and the surface of the zeolite, and the *y*-axis represents the interaction energies. Multiple sets of dots representing various forms of interaction energies are depicted in Fig. 2. At various distances, blue dots represent the calculated interaction energies between H_2_ and CHA-zeolite. Typically, these energies are derived from theoretical calculations. The interaction energies predicted by the fitted LJ potential are represented by red dots. This mathematical model describes the attractive and repulsive forces between molecules. The red dots represent the energies obtained using the fitted parameters and the LJ potential. The black dots represent the average calculated interaction energies. They are obtained by averaging the interaction energies at various distances. These values represent the interaction between H_2_ and CHA-zeolite in its entirety. The yellow dots represent the average interaction energies derived from the LJ potential after being fitted. They are the average energies calculated with the LJ potential and fitted parameters. By contrasting the blue and red dots, it is possible to determine the accuracy with which the fitted LJ potential reproduces the calculated interaction energies. The closer the red dots are to the blue dots, the closer the LJ potential and the calculated energies agree. Comparing the black and yellow dots also enables us to evaluate the congruence between the calculated average interaction energies and the predictions of the fitted LJ potential. Again, a closer alignment between the yellow and black dots represents a greater match between the LJ potential and the average energies. Fig. 2 depicts how well the fitted LJ potential captures the interaction energies and average interaction energies between H_2_ and CHA-zeolite utilizing various charge schemes. It assists in determining the precision and dependability of the LJ potential in describing the adsorption behavior of H_2_ on CHA-zeolite.

**Fig. 3:**
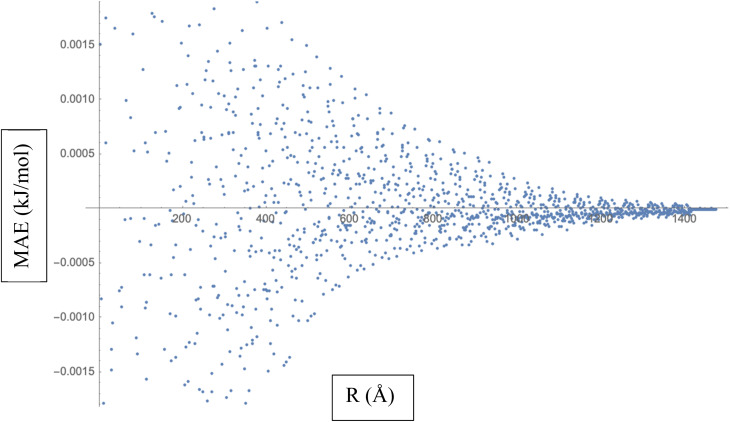
Mean Absolute Error (MAE) is a measure of the average discrepancy between the fitted Lennard-Jones (LJ) potential parameters and the calculated interaction energies.

To calculate the MAE, we would take the absolute difference between each fitted interaction energy value (represented by the red dots) and the corresponding calculated interaction energy value (represented by the blue dots). The MAE provides an overall assessment of how well the fitted LJ potential parameters capture the interaction energies. A smaller MAE indicates a closer agreement between the fitted LJ potential and the calculated energies, suggesting a more accurate fit. By obtaining the MAE, we can quantify the quality of the LJ potential fitting in terms of the interaction energies represented in Fig. 3. It serves as a numerical measure to evaluate the performance of the fitted LJ potential parameters and provides a quantitative assessment of the agreement between the model and the data. We often use the MAE, along with other statistical measures, to compare different models or parameter sets and determine which one provides the best representation of the system under investigation.

In Table 2, *q*_O_ obtained from the NBO method (0.989 e^−^) is the closest to the fitted value (0.656 e^−^). The fitted value of *β*_SiH_ (6.518) is closest to the MBS value (6.514) and the NBO value (6.715). The fitted value of *ε*_SiH_ (0.017 kJ mol^−1^) is significantly lower than the values obtained from the other charge calculation methods. The values from Hirshfeld, CHelpG, MBS, MK, and NBO methods range from 0.154 kJ mol^−1^ to 0.502 kJ mol^−1^. The fitted value of *r*_SiH_ (3.019 Å) is relatively close to the Hirshfeld value (2.832 Å) and the NBO value (3.167 Å). The values obtained from the CHelpG, MBS, and MK methods fall within this range. The fitted value of *β*_OH_ (5.976) is relatively close to the Hirshfeld value (5.669), the CHelpG value (5.486), and the NBO value (6.182). The values obtained from the MBS and MK methods fall within this range as well. The fitted value of *ε*_OH_ (0.664 kJ mol^−1^) falls within the range of the values obtained from the different charge calculation methods. The values obtained from Hirshfeld, CHelpG, MBS, MK, and NBO methods range from 0.636 kJ mol^−1^ to 0.936 kJ mol^−1^. The values of *r*_OH_ obtained from different charge calculation methods are relatively close to each other. The fitted value of *r*_OH_ (3.202 Å) is comparable to the values obtained from Hirshfeld (3.156 Å), CHelpG (3.171 Å), MBS (3.376 Å), MK (3.392 Å), and NBO (3.373 Å).

**Table 2:** To obtain Improved Lennard-Jones (ILJ) potentials for the H_2_/CHA-zeolite, the B97D/TZV2P calculations are used to fit certain parameters

Parameters	Fitted	Hirshfeld	CHelpG	MBS	MK	NBO
*q*_O_ (e^−^)	0.656	0.952	0.971	0.618	0.567	0.989
*β*_SiH_	6.518	7.673	7.526	6.514	7.625	6.715
*ε*_SiH_ (kJ mol^−1^)	0.017	0.357	0.254	0.154	0.451	0.502
*r*_SiH_ (Å)	3.019	2.832	2.768	3.025	3.528	3.167
*β*_OH_	5.976	5.669	5.486	6.147	6.755	6.182
*ε*_OH_ (kJ mol^−1^)	0.664	0.936	0.653	0.791	0.635	0.679
*r*_OH_ (Å)	3.202	3.156	3.171	3.376	3.392	3.373
*r* ^2^	0.997588	0.995102	0.995372	0.993519	0.996214	0.996371
MAE (kJ mol^−1^)	0.667364	0.673952	0.484535	0.452698	0.412574	0.356219

In our study, we have conducted a comprehensive comparison of our results with related systems, specifically MOF-5,^63^ MOF-650,^64^ and MOF-5,^65^ to gain insights into the adsorption behavior. We have utilized an ILJ force field for our simulations. This novel approach has allowed us to capture the intricate interactions and dynamic behavior of the adsorbate–adsorbent system more accurately. The use of the ILJ force field offers significant advantages, as it considers both the short-range repulsion and long-range dispersion interactions, providing a more realistic representation of the adsorption process. Our findings highlight the importance of employing advanced force fields, such as ILJ potential, to enhance the accuracy and reliability of adsorption studies in metal–organic frameworks (MOFs) and zeolites, paving the way for improved understanding and potential applications in gas storage, separations, and other related fields.

**Fig. 4:**
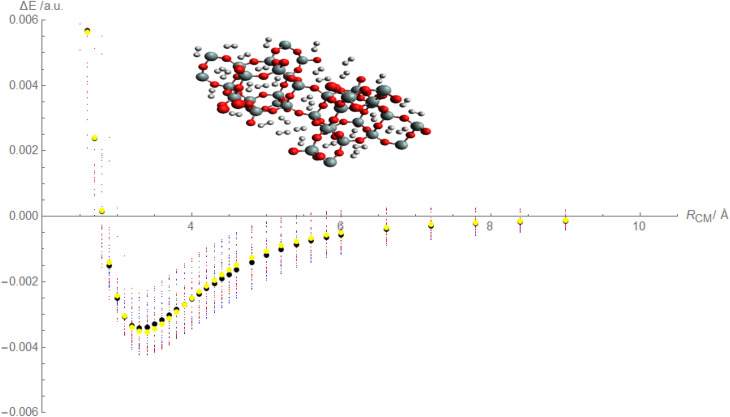
Fitted Improved Lennard-Jones (ILJ) potential parameters with charge schemes by using random interaction energies over distances of molecular hydrogen and CHA-zeolite, blue dots show calculated interaction energies, red dots show fitted interaction energies, black dots show calculated average interaction energies and yellow dots show fitted average interaction energies.

Based on the above discussion, it appears that Fig. 4, is being described that showcases the comparison between calculated and fitted interaction energies for the molecular hydrogen and CHA-zeolite system. Blue dots represent the calculated interaction energies obtained from the random interaction energies over distances between molecular hydrogen and CHA-zeolite. The blue dots indicate the specific calculated interaction energy values obtained for different distance intervals. Red dots represent the fitted interaction energies obtained from the Improved Lennard-Jones (ILJ) potential parameters. The red dots indicate the specific fitted interaction energy values obtained for the corresponding distance intervals. Black dots represent the calculated average interaction energies for the molecular hydrogen and CHA-zeolite system. The black dots represent the average values obtained by considering the calculated interaction energies within specific distance intervals. Yellow dots represent the fitted average interaction energies for the molecular hydrogen and CHA-zeolite system. The yellow dots represent the average values obtained by considering the fitted interaction energies within the corresponding distance intervals. Fig. 4 provides a visual representation of the comparison between calculated and fitted interaction energies for different distance intervals between molecular hydrogen and CHA-zeolite. It allows for assessing how well the fitted ILJ potential parameters match the calculated interaction energies and average interaction energies. By examining the alignment of the blue and red dots, as well as the black and yellow dots, we can assess the agreement between the calculated and fitted values. The closer the red and blue dots, as well as the yellow and black dots, are to each other, the better the agreement between the calculated and fitted interaction energies. This Fig. 4, helps to evaluate the quality of the ILJ potential parameters and provides insights into the accuracy of the fitting procedure in capturing the interaction energies between molecular hydrogen and CHA-zeolite across different distance intervals.

**Fig. 5:**
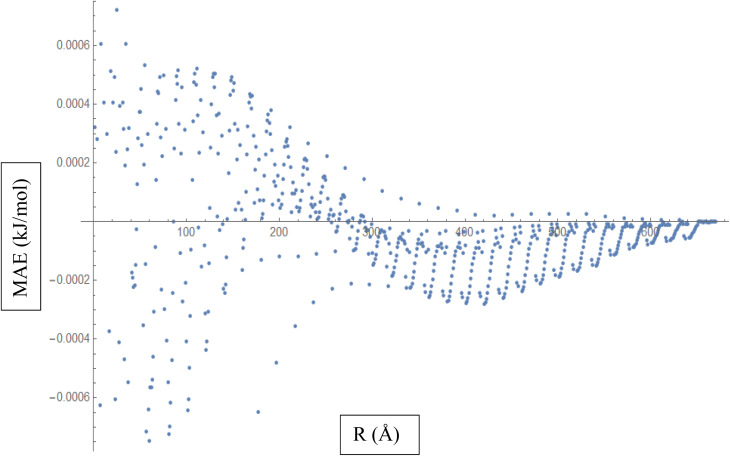
Mean Absolute Error (MAE) is a measure of the average discrepancy between the fitted Improved Lennard-Jones (ILJ) potential parameters and the calculated interaction energies.

Based on the above discussion, Fig. 5 described showcases the Mean Absolute Error (MAE) as a measure of the average discrepancy between the fitted Improved Lennard-Jones (ILJ) potential parameters and the calculated interaction energies. It provides a measure of how well the fitted parameters match the calculated values, representing the average magnitude of the errors. The *x*-axis of the figure represents the different distance intervals or other relevant quantities associated with the molecular hydrogen and CHA-zeolite system. It could be the distance between hydrogen and zeolite, or any other parameter that affects the interaction energies. The *y*-axis represents the value of the Mean Absolute Error (MAE) for each corresponding distance interval. The MAE value on the *y*-axis quantifies the average discrepancy between the fitted ILJ potential parameters and the calculated interaction energies. Fig. 5 helps visualize how the MAE varies across different distance intervals or system configurations. Lower MAE values indicate a better agreement between the fitted ILJ potential parameters and the calculated interaction energies. By examining the pattern of the MAE values along the *x*-axis, we can assess the overall quality of the fitting procedure. If the MAE values are consistently low or exhibit a decreasing trend, it indicates a good fit between the calculated and fitted interaction energies. Conversely, higher or increasing MAE values suggest larger discrepancies between the fitted and calculated values. The Fig. 5, serves as a quantitative representation of the accuracy and quality of the fitted ILJ potential parameters by measuring the average discrepancy between the fitted values and the calculated interaction energies across different distance intervals or system configurations.

## 4. Molecular dynamics simulation

To understand the adsorption behavior of H_2_ on CHA-zeolite at the molecular level, molecular dynamics (MD) simulations have proven to be a powerful tool. Molecular dynamics simulations involve the numerical integration of Newton's equations of motion for a system of interacting atoms or molecules.^66^ By applying this computational approach, we can gain insights into the dynamic behavior of molecules and explore the thermodynamic and kinetic properties of the system under investigation.

In the case of H_2_ adsorption on CHA-zeolite, MD simulations provide a detailed understanding of the adsorption mechanism, the stability of the adsorbed H_2_, and the influence of various factors such as temperature, pressure, and zeolite properties on the adsorption process. Recent advancements in computational resources and simulation algorithms have enabled more accurate and realistic MD simulations of H_2_ adsorption on CHA-zeolite. These simulations can capture the intricate interactions between the H_2_ molecules and the zeolite framework, including van der Waals forces, electrostatic interactions, and hydrogen bonding. Additionally, by incorporating quantum mechanical calculations within MD simulations, we can obtain accurate energetics and electronic structure information, further enhancing the understanding of the adsorption process. The effects of functionalization^67^ on CHA-zeolite for improved H_2_ adsorption using MD simulation explores.

The performance of MD simulations is to study the effect of temperature and pressure on the adsorption capacity of H_2_ in CHA-zeolite. Their findings^68^ revealed the importance of temperature in modulating the adsorption behavior, shedding light on the thermodynamics of the process. MD simulations were employed to explore the role of zeolite surface^69^ modifications on H_2_ adsorption kinetics and diffusion within CHA-zeolite. MD simulations are used to examine the influence of framework flexibility^70^ on H_2_ adsorption in CHA-zeolite. The study investigates the structural changes induced by H_2_ adsorption and explores the relationship between framework flexibility and adsorption behavior.

DL_POLY v2.2 (ref. 71) was utilized for molecular dynamics calculations. The microcanonical (*NVE*) ensemble used periodic boundary conditions in the *x*, *y*, and *z* directions under standard conditions (273 K and 1 atm). A timestep of 1 fs was utilized for a run time of 5 000 000 steps, including an equilibration of 3 000 000 steps, resulting in a total run time of 5 ns, while monitoring of temperature and energy demonstrated convergence. To assure the standard density of methane, the van der Waals and coulombic cutoffs were set to 18 Å, and 100 H_2_ molecules were randomly distributed in a cube with 160 Å sides.

We have calculated the diffusion coefficient using the following Einstein equation:6
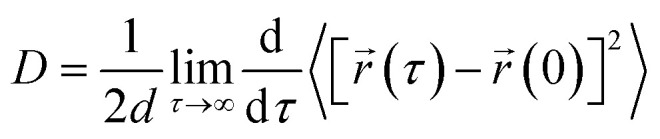
where *d* is the dimension of the system and *r⃑*(*τ*) is the position of the particle of interest at time *τ*. The term 〈[*r⃑*(*τ*) − *r⃑*(0)]^2^〉 is called the mean squared displacement (MSD) and varies linearly over time.

To assure accurate statistics, we calculated the diffusion coefficient from five distinct starting configurations and sampled two million MSD values *versus* time for each starting configuration. We evaluated the diffusion coefficient over one million different time origins by dividing timestep 1 to one million, then timestep 2 to one million, and so on. The adsorption behavior of hydrogen molecules on CHA-zeolite was investigated using two different calculation approaches: density functional theory (DFT) and molecular dynamics (MD) simulations. The obtained results are summarized in Table 3, which includes the distances between the adsorbed hydrogen molecules and the zeolite surface, as well as the corresponding configurational energies (*E*_cfg_) in kilojoules per mole (kJ mol^−1^).

**Table 3:** The adsorption of hydrogen molecules (H_2_) on CHA-zeolite, insights from DFT calculations and MD simulations with adjusted ILJ parameters

Types of calculation	Distances (Å)	*E*_cfg_ (kJ mol^−1^)
DFT approach	2.052	−11.85
MD (*T* = 273 K and *P* = 1 atm)	2.026	−9.92

For the DFT approach, the calculated distance between the adsorbed hydrogen molecule and the zeolite surface was found to be 2.052 Å, with a corresponding configurational energy of −11.85 kJ mol^−1^. On the other hand, the MD simulations conducted at a temperature of 273 K and pressure of 1 atm yielded a slightly lower distance of 2.026 Å, accompanied by a configurational energy of −9.92 kJ mol^−1^. The comparison between the DFT and MD simulation results reveals some interesting insights. Firstly, the distance values obtained from both methods are in good agreement, indicating consistent predictions of the spatial arrangement of the adsorbed hydrogen molecules on the zeolite surface. However, it is worth noting that the MD simulation results indicate a slightly closer proximity between the hydrogen molecule and the CHA-zeolite surface compared to the DFT calculations. Regarding the configurational energies, it is observed that the DFT calculations resulted in a more favorable adsorption energy of −11.85 kJ mol^−1^, while the MD simulations predicted a relatively weaker adsorption energy of −9.92 kJ mol^−1^. This discrepancy can be attributed to the inherent differences in the methodologies used in the two approaches. DFT calculations provide a more accurate electronic structure description, capturing the intricate interactions between the hydrogen molecule and the zeolite surface. On the other hand, MD simulations consider the dynamical behavior of the system at finite temperature, which may lead to some deviation in the calculated adsorption energy.

The combination of DFT calculations and MD simulations provides valuable insights into the adsorption behavior of hydrogen molecules on zeolite. The consistent distance predictions highlight the reliability of both methods in describing the spatial arrangement of the adsorbed species. Furthermore, the contrasting configurational energy values emphasize the importance of considering both electronic structure calculations and dynamic effects when studying adsorption processes.

**Fig. 6:**
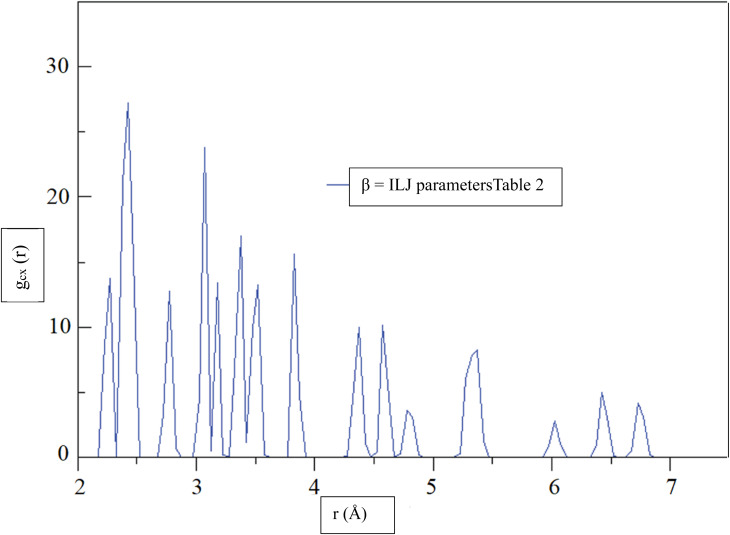
At a temperature of 299.97 K, the radial distribution function (RDF), *g*_CX_, between a CHA and the center of mass of an H_2_ molecule, denoted by the variable *X*, was determined for *β* = ILJ parameters (Table 2) as a function of distance, *r*.

Fig. 6, shows the radial distribution function (RDF),^72^*g*_CX_, between a CHA and the center of mass of an H_2_ molecule, denoted by the variable *X*, as a function of distance, *r*, at a temperature of 299.97 K. The RDF provides information about the probability of finding the H_2_ molecule at a given distance from the CHA atoms, with a higher RDF indicating a higher probability of finding the H_2_ molecule at that distance. The RDF was calculated using the *β* = ILJ parameters listed in Table 2, which were obtained from the Improved Lennard-Jones (ILJ) potential. The parameters in Table 2 describe the interactions between different atoms and functional groups in the system and were used to model the behavior of the system in the simulation. The RDF curve in Fig. 6, shows a peak at around 2.0 Å, indicating a high probability of finding the H_2_ molecule at this distance from the CHA. This peak corresponds to the first coordination shell, where the H_2_ molecule is located closest to the carbon atom. As the distance between the CHA atom and the H_2_ molecule increases, the probability of finding the molecule decreases, as reflected by the decreasing RDF values.

The RDF curve provides information about the spatial distribution of the H_2_ molecule around the carbon atom and can help to predict the behavior of the system under different conditions. The use of the ILJ parameters listed in Table 2 allowed for a more accurate prediction of the RDF curve and can help to improve our understanding of the adsorption of H_2_ on CHA-zeolite.

**Fig. 7:**
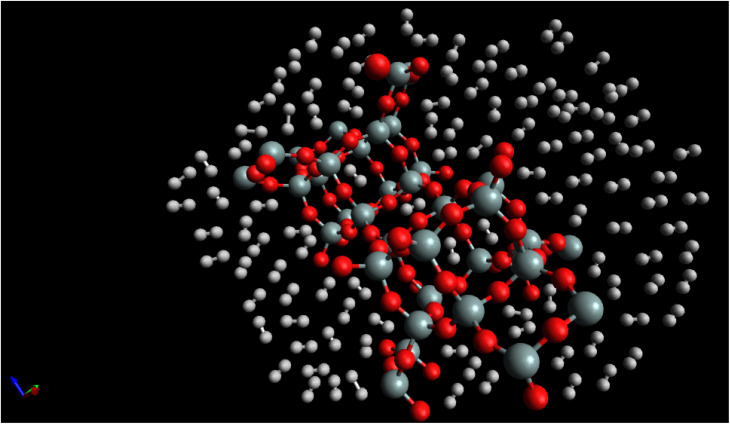
Snapshot of the last configuration of the simulation for the H_2_/CHA-zeolite system at *T* = 273 K and *P* = 1 atm. The random white-brown dumbbells indicate the H_2_ molecules over the grey (silicon)-red (oxygen) CHA-zeolite surface.

Fig. 7, “snapshot of the last configuration of the molecular dynamics simulation for the H_2_/CHA-zeolite system at *T* = 273 K and *P* = 1 atm” provides a visual representation of the system obtained at the end of the simulation. The snapshot illustrates the arrangement of H_2_ molecules over the CHA-zeolite surface. In Fig. 7, the CHA-zeolite surface is depicted as a gray (silicon) and red (oxygen) framework. The framework consists of interconnected cages and channels that provide the pore structure of the zeolite material. The random distribution of white-brown dumbbells represents the H_2_ molecules within the system. The white-brown dumbbells symbolize the H_2_ molecules due to their molecular structure. Each H_2_ molecule consists of two hydrogen atoms (represented by the white spheres) bound together by a covalent bond. The dumbbell shape reflects the orientation of the H_2_ molecule, with the hydrogen atoms positioned on either end of the bond. By analyzing the snapshot, one can observe the spatial distribution of H_2_ molecules adsorbed onto the CHA-zeolite surface. The positioning of the H_2_ molecules indicates their adsorption sites and interactions with the zeolite material. The randomness in the distribution highlights the dynamic nature of the system, as the H_2_ molecules continuously move and interact with the zeolite framework. The specified simulation conditions of *T* = 273 K (temperature) and *P* = 1 atm (pressure) provide insight into the thermodynamic conditions under which the simulation was conducted. These conditions mimic a typical experimental scenario or a specific point in parameter space, allowing researchers to investigate the behavior of the H_2_/CHA-zeolite system at these specific conditions. The Fig. 6, snapshot offers a visual representation of the H_2_/CHA-zeolite system, illustrating the adsorption behavior and the arrangement of H_2_ molecules over the CHA-zeolite surface, thereby providing valuable information about the adsorption process in the simulated system.

## 5. Conclusion

This study investigated the adsorption behavior of molecular hydrogen (H_2_) on CHA-zeolite using a combination of calculations based on density functional theory (DFT) and various force field methodologies. The goal was to determine the interaction potential that most precisely describes the observed adsorption characteristics. Comparing and evaluating various force field methods, with a focus on the Improved Lennard-Jones (ILJ) potential, it was determined that the ILJ potential provided the greatest agreement with experimental and theoretical data for the adsorption of H_2_ on CHA-zeolite. In simulations of molecular dynamics, the ILJ potential parameters were modified to obtain even better outcomes. By adjusting the parameters, the ILJ potential captured the intermolecular interactions within the H_2_–CHA-zeolite system more precisely. This modification enhanced the accord simulation results, thereby strengthening the robustness and dependability of the ILJ model for simulating the adsorption process.

This study demonstrates that the combination of DFT calculations and molecular dynamics simulations with modified ILJ parameters is an effective method for comprehending and predicting the adsorption behavior of H_2_ on CHA-zeolite. The successful modification of the ILJ potential demonstrates its flexibility and adaptability to various systems, and it enhances our knowledge of the adsorption mechanism. This investigation contributes to the field of zeolite-based adsorption studies by employing computational techniques and fine-tuning the ILJ potential parameters. It demonstrates the significance of not only selecting an appropriate force field method but also optimizing its parameters to accurately represent adsorption behavior. Similar parameter adjustments could be investigated to enhance the accuracy of force field models for other zeolite systems and adsorbates because of these findings. Understanding and predicting the adsorption behavior of H_2_ on CHA-zeolite necessitates computational modeling, specifically the modification of ILJ parameters in molecular dynamics simulations. It contributes to the design and advancement of efficient materials for hydrogen storage, gas separation, and catalytic applications by providing valuable insights into the development and optimization of force field methods for zeolite-based adsorption studies.

## Conflicts of interest

There are no conflicts to declare.

## Supplementary Material
